# Supplementary material for: Exploring Conformational Transitions of Adenine RNA Dimer via Machine Learning Potentials
Source: J Chem Theory Comput. 2026 Aug 17;22(17):9199–213. doi: 10.1021/acs.jctc.6c01213 (PMC13564357; doi:10.1021/acs.jctc.6c01213)
Supplement: Supplementary file 1 [file ct6c01213_si_001.pdf]

Supporting Information

Exploring Conformational Transitions of Adenine RNA Dimer via Machine Learning Potentials

Leonardo Medrano Sandonas[1,*], Macarena Tolmos Nehme[2], Luis Fernando Cofas-Vargas[3], Gustavo E. Olivos-Ramirez[4], Gianaurelio Cuniberti[1,5,6], Simón Poblete[7,8,*], Adolfo B. Poma[4,*]

[1]Institute for Materials Science and Max Bergmann Center of Biomaterials, TUD Dresden University of Technology, 01062, Dresden, Germany
[2]Universidad Nacional de Ingeniería, Av. Túpac Amaru 210, Rímac, Lima 15333, Peru
[3]Departamento de Química, Universidad Autónoma Metropolitana-Iztapalapa, Mexico City C.P. 09310, Mexico
[4]Department of Biosystems and Soft Matter, Institute of Fundamental Technological Research, Polish Academy of Sciences, ul. Pawińskiego 5B, 02-106, Warsaw, Poland
[5]Cluster of Excellence CARE, TUD Dresden University of Technology and RWTH Aachen, 01062, Dresden, Germany
[6]Cluster of Excellence CeTI, TUD Dresden University of Technology, 01062, Dresden, Germany
[7]Facultad de Ingeniería, Universidad San Sebastián, bellavista 7, 8420524, Santiago, Chile
[8]Centro BASAL Ciencia & Vida, Universidad San Sebastián, av. del valle norte 725, 8580702, Santiago, Chile

1 RNA simulation details

Here we will enumerate and describe the data generated for the ML analysis.

1.1 ApA-TREMD

It corresponds to the Temperature-Replica Exchange MD simulation of a Adenine-Adenine dinucleotide monophosphate. That is, two nucleotides connected through the backbone where the first one does not have a phosphate group. In consequence, a single positive ion must be included to keep the neutrality of the whole system. The DMNP is solvated in 1080 water molecules and a Sodium Ion in a cubic box of length $L = 32.1719$Å using periodic boundary conditions. The initial condition was taken from an A-form duplex, and then energy-minimized with a steepest-descent algorithm, to be relaxed with a short NpT simulation for 1 ns.

Following the notation of the work of Hayatshahi et al.[1], the force field employed was ff14 (Amber99 with parmbsc9 and χOL corrections) with a TIP3P water model. Following the same reference, we used a Temperature-Replica Exchange Molecular Dynamics simulation for 500 ns, which was reported to sample the conformation space satisfactorily. The MD was integrated using the leapfrog method with a timestep of 0.002 ns, while temperature was kept constant with a velocity rescaling with a stochastic term thermostat with a constant of 0.1 ps. The temperatures were 280, 285.8, 291.7, 297.7, 303.9, 310.1, 316.6, 323.1, 329.8, 336.6, 343.5, 350.6, 357.9, 365.3, 372.8, 380.5, 388.4 and 396.4 K where the exchange acceptance was between 20 and 30%. We checked that the temperature space was thoroughly and frequently visited by each replica. Finally, we selected the simulation at T=297.715K .

1.1.1 Data files

For the selected simulation, we have deployed the trajectory in pdb and xyz formats, composed of snapshots saved every 50 ps, giving a total of 10,000 frames. The coordinates of the atoms were centered around the group of atoms composing the RNA molecule on each timestep. The rest of the files are in xvg format, which

is an ascii file where the data is stored in lines for each timestep. The data is extended along columns, whose content is detailed in the first commented lines in the file, starting with the characters "`@`" or "`#`".

An offline analysis of the forces was performed on the selected simulation. The file `cropped_force.xvg` contains the force on each atom, which are numbered according the pdb file. Each line contains the timestep in each column, followed by the three coordinates of the total force on every different atom. The first lines of the datafile, which begin with the character "`@`", detail this correspondence.

The energies of the whole system are also calculated, keeping track of different contributions. Taking into account the energy terms of the AMBER force field, it makes sense to decompose the energy between the groups of RNA and non-RNA atoms. The second is composed by the water molecules and the Na ion. These groups are labeled as `RNA` and `IWAT`, respectively. The energies are reported for the interactions between `RNA-RNA`, `RNA-IWAT` and IWAT-IWAT. Given the treatment of the electrostatic interactions, and additional term which is not possible to decompose under this approach is the Coulomb-reciprocal, which is also included for completeness. In order to provide more detail in the contributions of each of these terms, the xvg files are divided into bonded (only for intra-RNA interactions) and non-bonded, which have terms of Lennard-Jones and Coulomb in different columns on each timestep. The lines in the header of the file specify which interaction corresponds to each column. Nevertheless, the sum over the columns of an energy file, excluding the time, yield the total energy for bonded or non-bonded cases.

1.1.2 Stability of ApA centroids in MD simulation using MLPs

A visual and quantitative evaluation of the degree to which the sampled conformers from simulations resemble the five reference stacking conformations is provided by the structural overlays in Fig. S4. The RMSD values reported correspond to the frame with the lowest deviation from each reference structure, providing insight into the accuracy of sampled conformations. In general, the inverted conformation showed the best structural agreement across these four models, with RMSD values consistently around 0.12-0.24 Å. This means that this conformation was not only frequently sampled, as indicated by the population data, likely due to its stable stacking conformation. The A-form conformation resulted in lower RMSD values (approx. 0.15 Å in SO3LR follower by both models RNA-TB and RNA-DFT with 0.52 Å) and m4-8 (0.25 Å) respectively. The ladder and sheared conformations showed greater structural variability across the models and sampling schemes. The RMSD values for ladder was 1.30 Å for RNA-TB, 1.33 Å for RNA-DFT and 1.41 Å for SO3LR . The sheared conformation showed a comparable pattern, with RMSD values exceeding 1 Å for both RNA models. The anti-ladder conformation showed moderate agreement with the reference in all conditions. RMSD values were consistently in the range of 0.23-0.45 Å, indicating that this conformation is sampled with consistency and may represent a recurrent intermediate state between canonical and unstacked forms. SXThe SO3LR sampled significantly very close conformations of the ApA system. Either means that SO3LR over-fitted the A-form conformation or lacked sensitivity to capture alternative stacking arrangements.

1.1.3 Optimal selection of the number of clusters

The selection of the optimal number of clusters K was performed through a systematic sensitivity analysis of the TREMD reference trajectory over the range $K = 2$–10, evaluating four complementary internal validation criteria. The Davies–Bouldin Index (DBI), shown in Fig. S9(a), attains its global minimum at $K = 6$ (DBI = 0.94), indicating that this partition maximizes inter-cluster separation relative to intra-cluster dispersion. The mean silhouette coefficient $\langle S_i \rangle$, displayed in Fig. S9(b), reaches its maximum value of 0.42 at $K = 6$, remaining above the weak-structure threshold of 0.25 for all six clusters. For $K \geq 8$, multiple clusters exhibit $\langle S_i \rangle < 0.25$ and populations below 4%, indicating statistically poorly represented conformational states. The composite

score, Fig. S9(c), constructed from the normalized combination of the four criteria, is likewise maximized at $K = 6$, confirming the consensus optimum. The resulting cluster populations, Fig. S9(d), span the range 7.2%–38.9%, demonstrating a well-distributed partitioning of the conformational space without degenerate or marginally populated states.

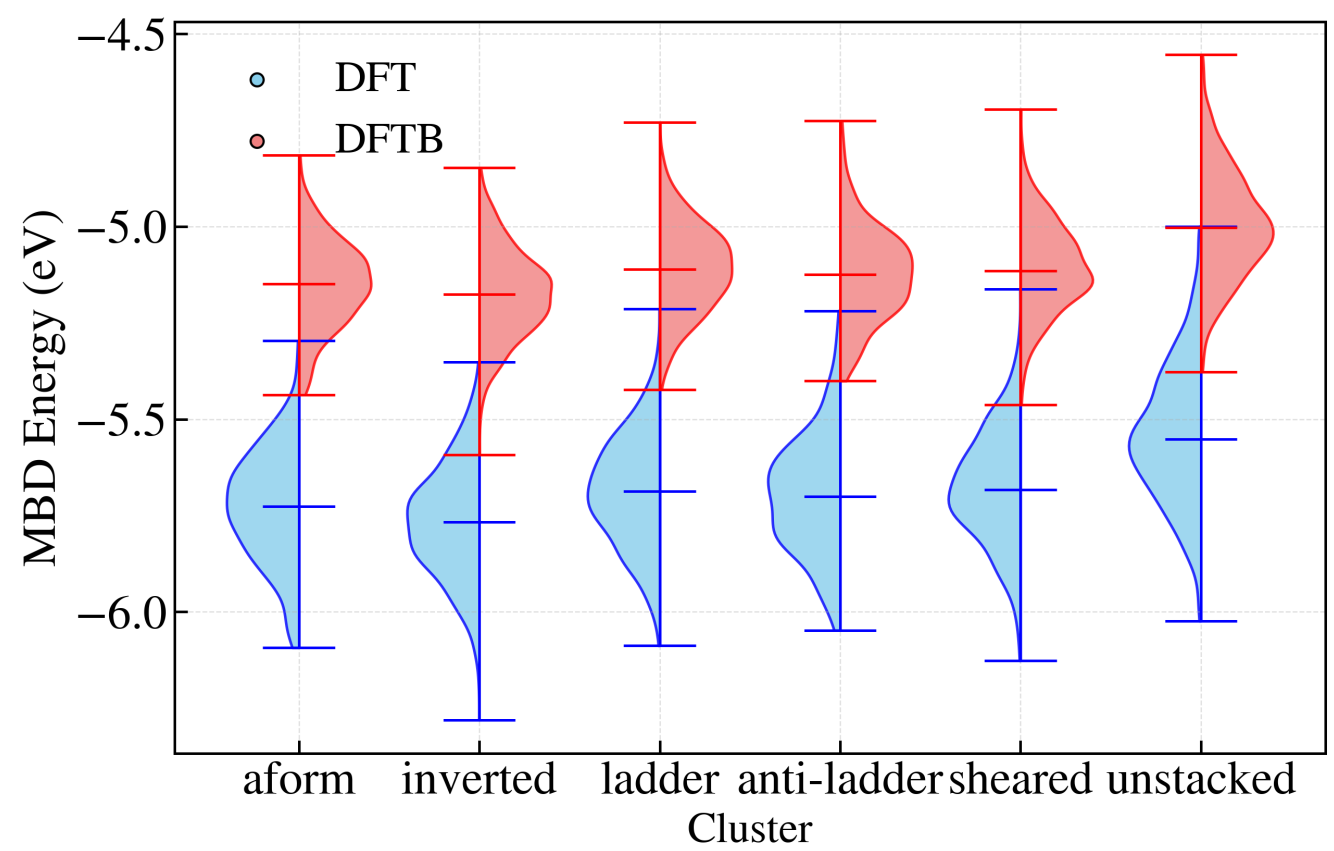

Figure S1 Distributions of the MBD energies obtained with DFT and DFTB for ApA structures containing 194 atoms. We have divided them per conformational cluster.

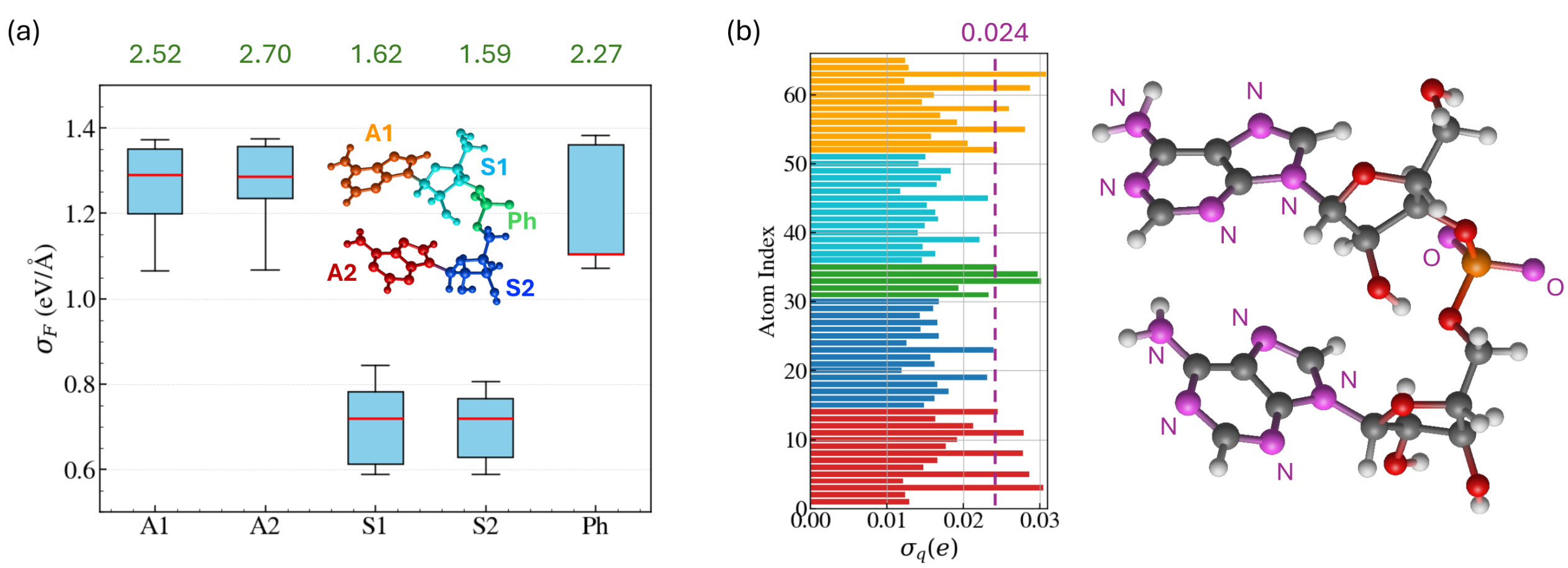

Figure S2 (a) Box plots of the standard deviation of atomic forces, σ_{F}, computed with DFTB for the ApA building blocks: adenine 1 (A1), adenine 2 (A2), sugar 1 (S1), sugar 2 (S2), and phosphate (Ph). Mean force values for each group are also shown on the top of box plots. (b) We show the variance of atomic charges, σ_{q}, computed with DFTB for all atoms in the ApA dimer. The atom index has been ordered with respect to the molecular groups defined in panel (a). In the inserted structure, we have highlighted in violet the atoms with $\sigma_{\mathrm{q}} > 0.024$e.

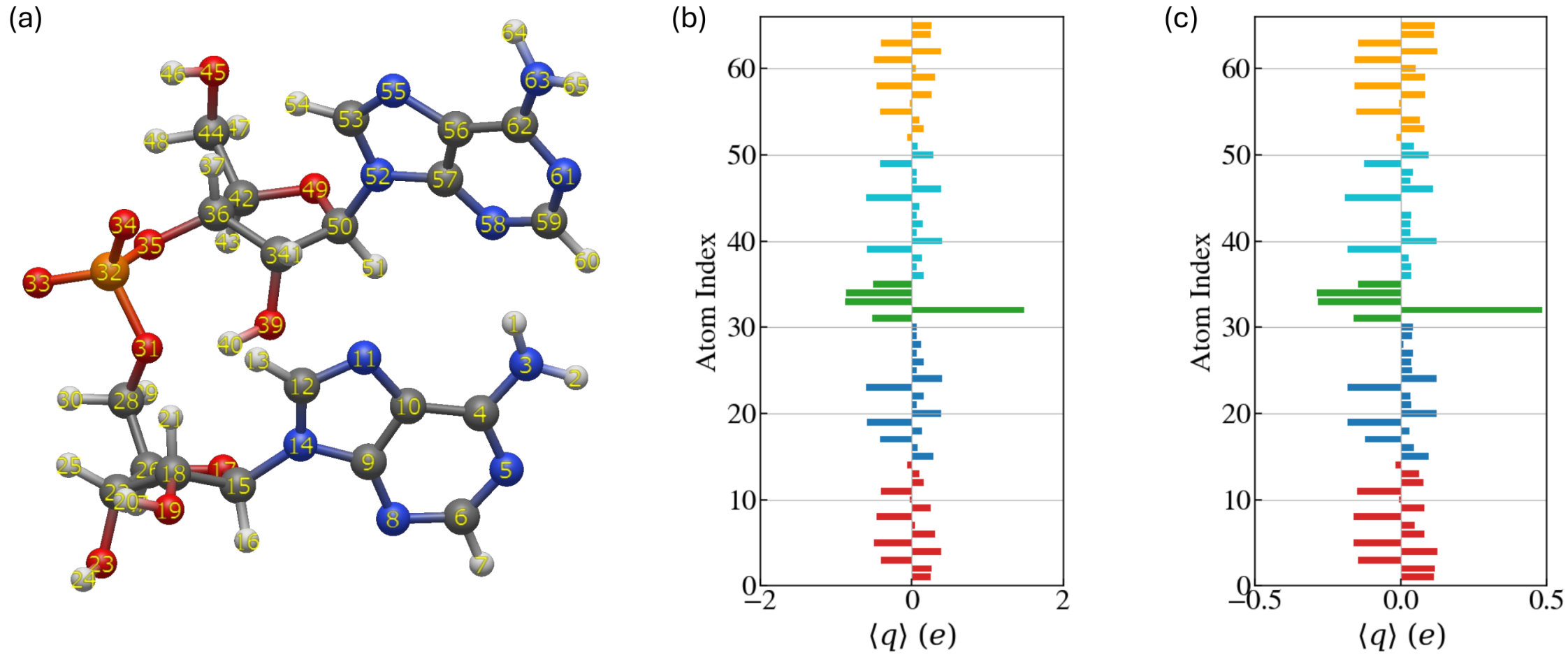

Figure S3 (a) ApA structure displaying the indices used for the analysis of atomic charges, q, in the Fig. 2 of the main text and Fig. S2 of the SI. We show the mean value of atomic charges, $\langle q \rangle$, computed by (b) DFT and (c) DFTB.

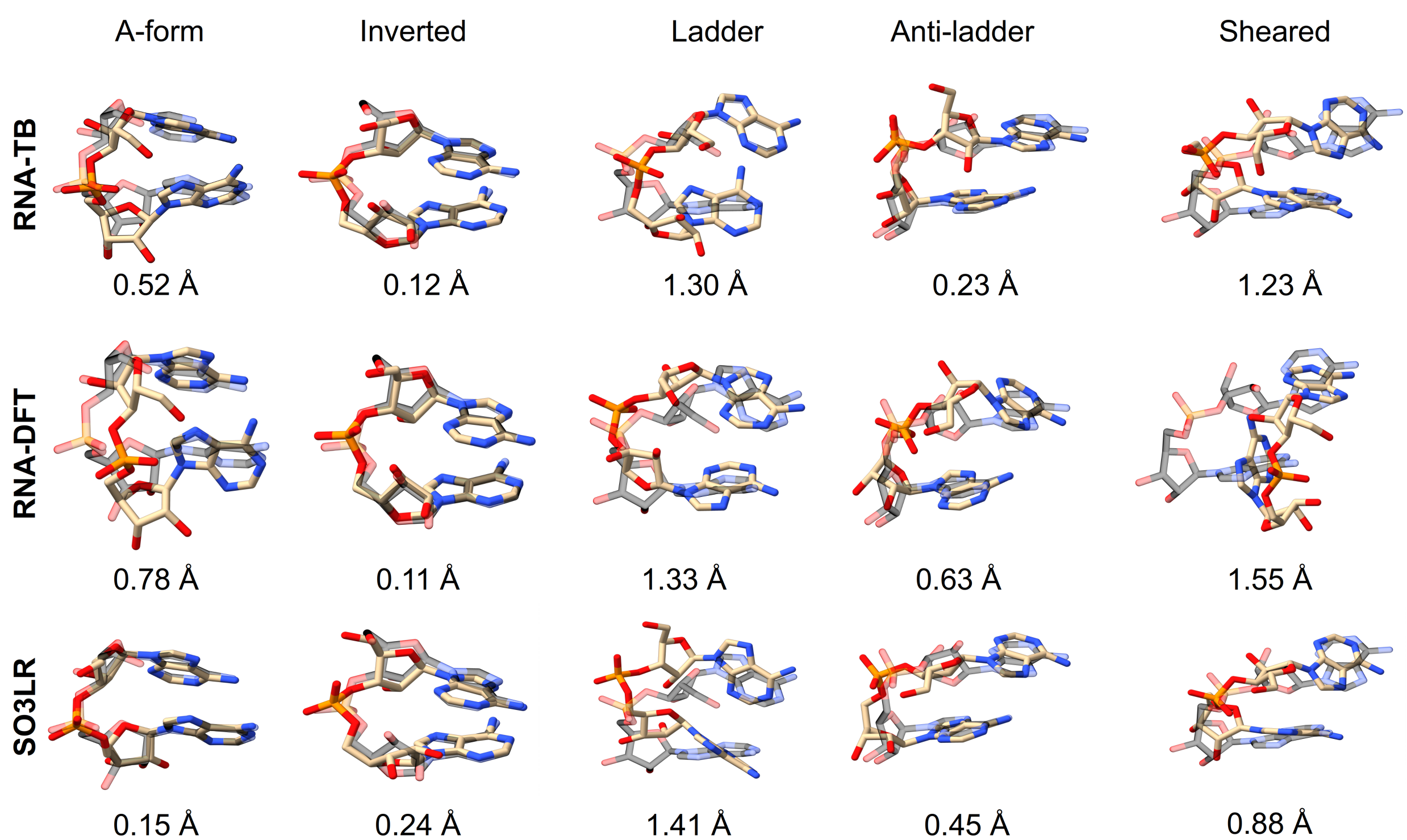

Figure S4 Overlay of representative ApA RNA dimer conformations with their corresponding reference structures for five stacked geometries: A-form, inverted, ladder, anti-ladder, and sheared. Transparent models indicate the reference structures, while opaque models represent the simulation frame with the lowest RMSD to each reference. Reported RMSD values (in Å) between representative frames and their respective reference conformations.

2 Additional validation of machine learning models

2.1 Hyperparameter optimization

Table S1 Evaluation of the performance of ML potentials trained on DFTB3+MBD (RNA-TB) and PBE0+MBD (RNA-DFT) property data for the ApA system. The equivariant MACE architecture was employed for model development. Mean absolute errors (MAEs) in the prediction of total energies E and atomic forces $\mathbf{F}_{\text{at}}$ are reported for all ApA conformations, as well as MAEs in $\mathbf{F}_{\text{at}}$ resolved by conformational cluster. The simulation time (in seconds) to perform an MD simulation at 300 K for 1 ps has also been added per model. Errors for energies and atomic forces are reported in mkcal/mol per atom and kcal/mol·Å, respectively.

Model	Hyperparameter			Full		error in F_{at} per cluster						time
	N_{int}	l_{max}	r_c	E	$\mathbf{F}_{\text{at}}$	A	I	L	AL	S	U	
RNA-TB	2	1	4.0	8.33	0.329	0.326	0.322	0.326	0.331	0.327	0.339	438.3
	2	2	4.0	7.65	0.303	0.300	0.297	0.300	0.306	0.302	0.313	828.4
	2	2	5.0	2.63	0.128	0.125	0.123	0.126	0.128	0.127	0.135	995.3
	1	1	5.0	16.88	0.746	0.739	0.741	0.739	0.761	0.745	0.752	251.0
RNA-DFT	2	1	4.0	9.64	0.321	0.319	0.315	0.318	0.323	0.320	0.328	407.5
	2	2	4.0	8.82	0.290	0.288	0.285	0.287	0.293	0.289	0.296	866.2
	2	2	5.0	3.32	0.132	0.130	0.128	0.130	0.133	0.131	0.137	869.7
	1	1	5.0	18.96	0.771	0.769	0.766	0.767	0.783	0.772	0.774	240.2

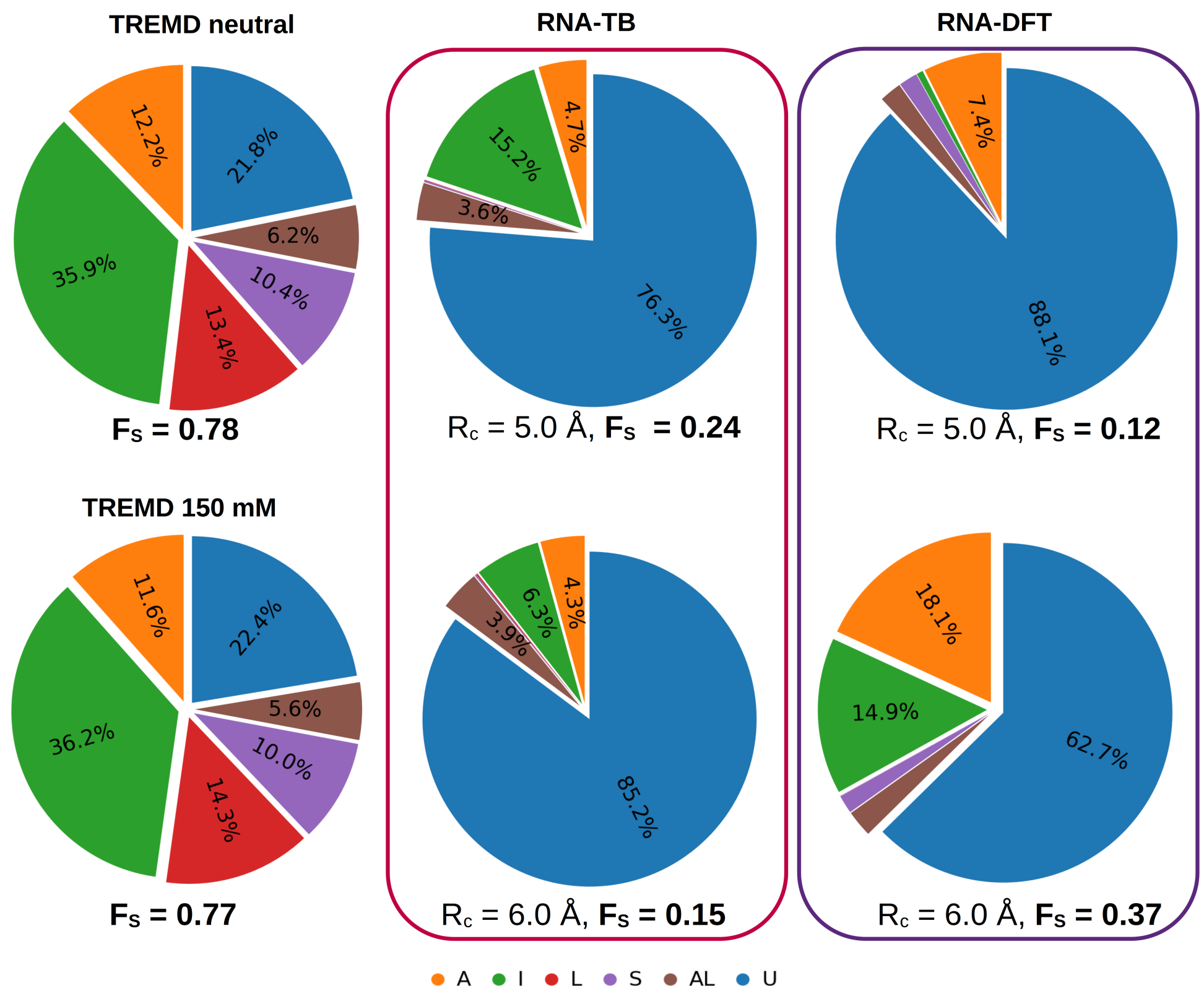

Figure S5 Comparison of stacking conformational ensembles and transition behavior for ApA dimers across models. Pie charts depict the relative populations of six conformational classes: A-form (A), inverted (I), ladder (L), anti-ladder (AL), sheared (S), and unstacked (U).

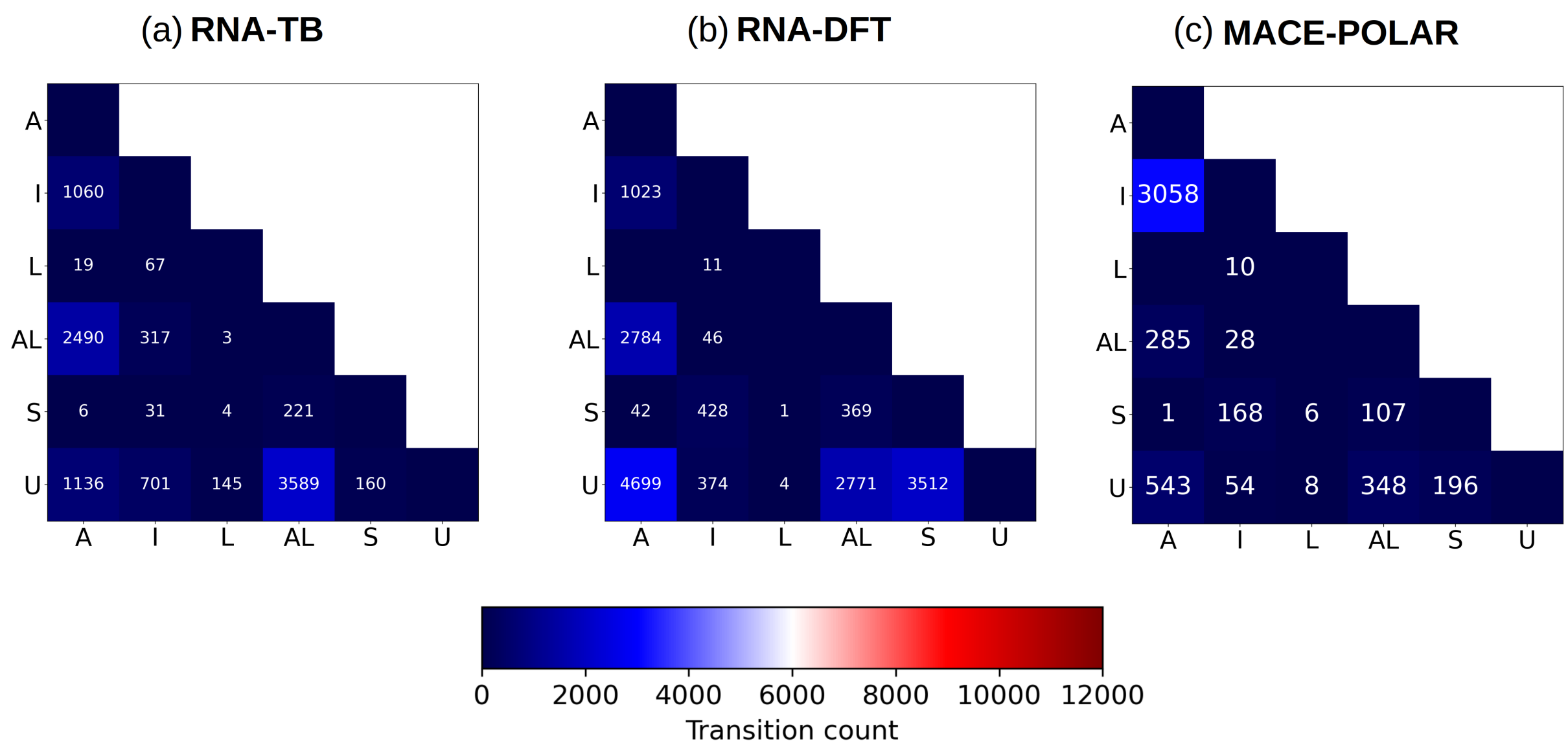

Figure S6 Transition behavior for ApA dimers across models. Stacking transition matrices show the number of transitions between stacking states for each model. Each panel corresponds to a different method, with transitions accumulated from three 10-ns simulations per model (sampled every 10 fs, yielding 10^6 frames). Cutoff radius of $R_c = 6$ Å for RNA-TB and $R_c = 5$ Å for RNA-DFT.

3 Additional structural analysis of MD trajectories

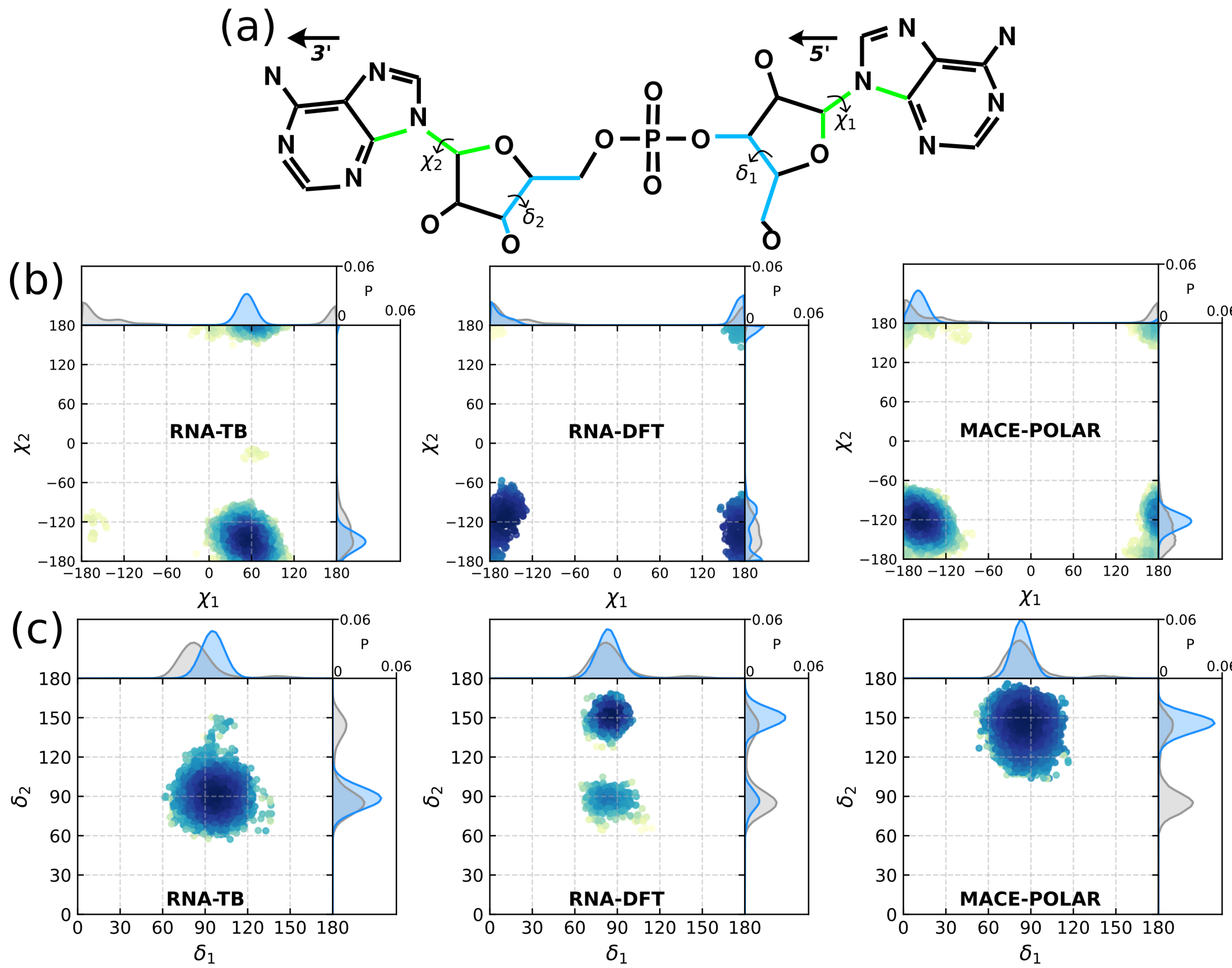

Figure S7 (A) Chemical structure of the ApA RNA dimer showing the definition of the χ and δ torsion angles for both nucleotides. (B) Two-dimensional distributions of χ_1 and χ_2 angles sampled during simulations using the RNA-TB (R_c = 6 Å), RNA-DFT (R_c = 5 Å), and MACE-POLAR models. (C) Two-dimensional distributions of δ_1 and δ_2 angles for the same models. The color scale represents the normalized probability of sampling each pair of torsional angles. The reference TREMD distributions are shown in gray color.

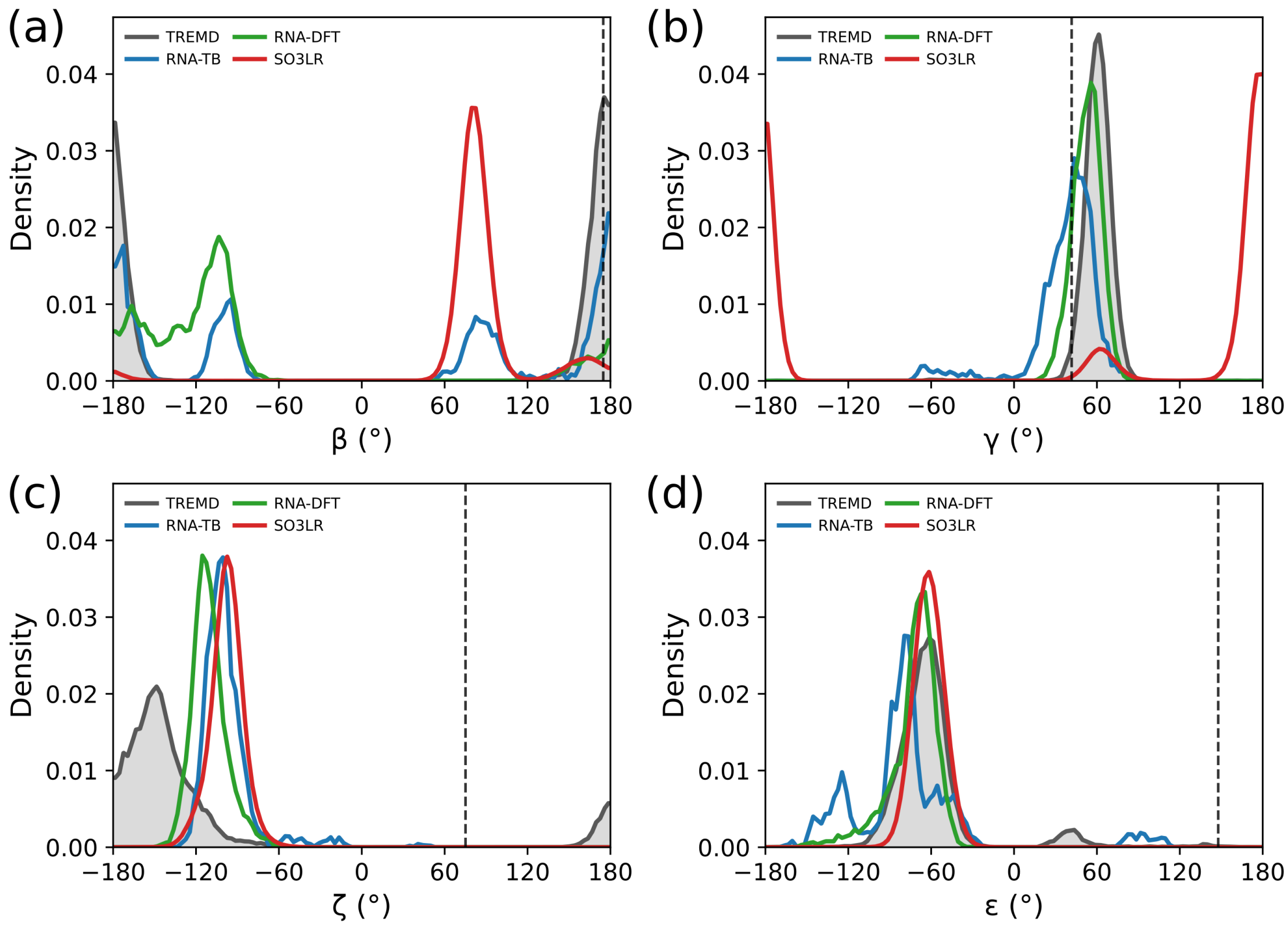

Figure S8 Single distributions for the reference TREMD data and the RNA-TB (R_c = 5 Å), RNA-DFT (R_c = 6 Å), SOL3R models. Vertical dashed lines indicate the experimental values for A-form[2].

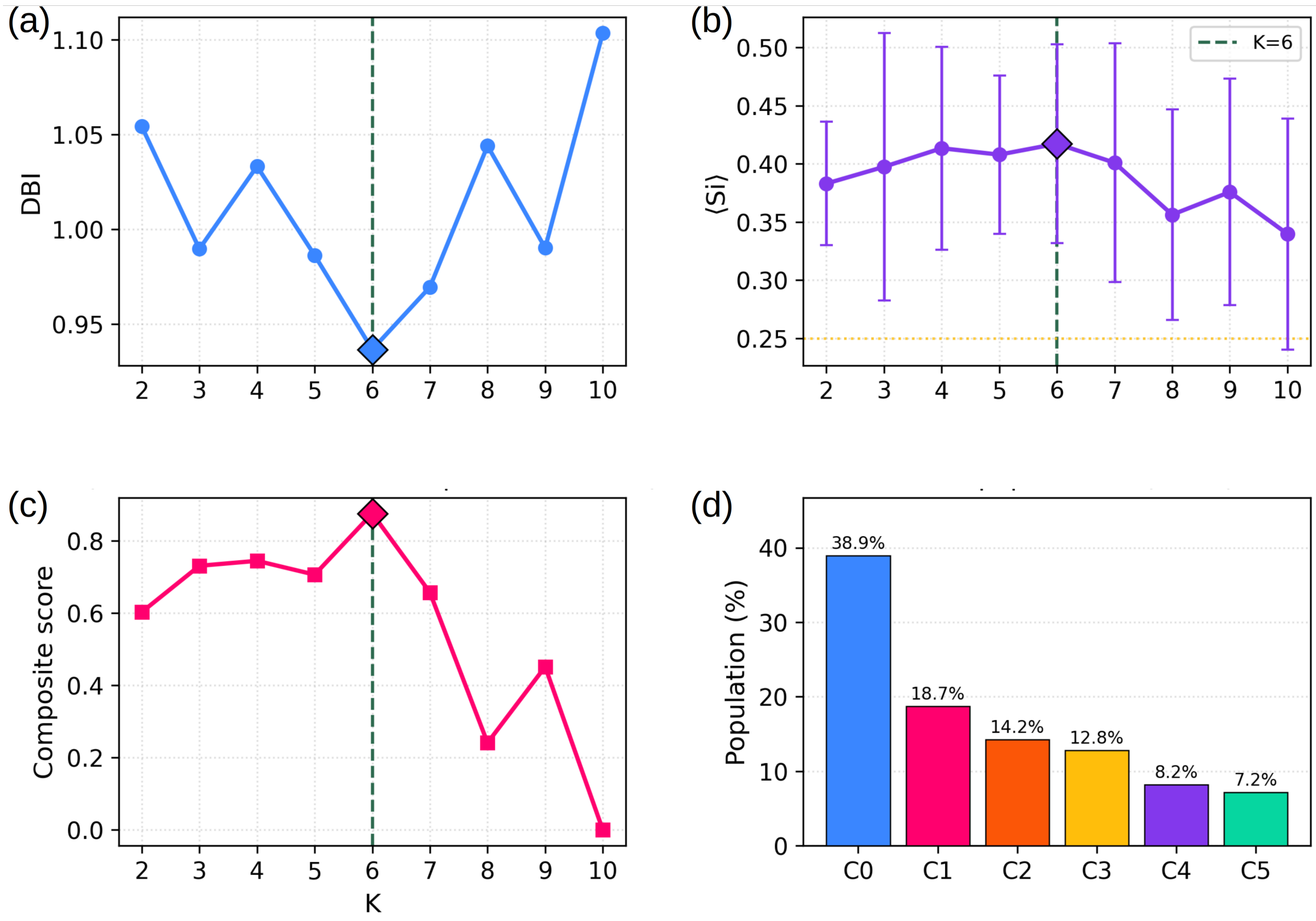

Figure S9 Optimal K selection for K-means clustering of the ApA dinucleotide TREMD trajectory. (**a**) Davies–Bouldin Index (DBI), with global minimum at $K = 6$ (DBI = 0.94). (**b**) Mean silhouette coefficient $\langle S_i \rangle \pm$ SD; dotted lines indicate the weak- (0.25) and reasonable-structure (0.50) thresholds[?]. (**c**) Composite score, maximized at $K = 6$. The dashed green vertical line marks the selected optimum in panels (a)–(c). (**d**) Cluster populations for $K_{\mathrm{opt}} = 6$, yielding six conformational states with populations ranging from 7.2% (C5) to 38.9% (C0).

Table S2 Atomic nomenclature of RNA bases and sugar-phosphate backbone

**Component**	**Atomic nomenclature**
Base	N1, C2, N3, C4, C5, C6, N7, C8, N9
Backbone	P, OP1, OP2, O5', C5', C4', O4', C3', O3', C2', O2', C1'

Table S3 Hellinger distance sensitivity to histogram discretization. Values are reported for each dihedral torsion and machine learning potential as the mean Hellinger distance (H_{mean}) and the corresponding error of the mean).

**Dihedral angle**	**Model**	H_{mean}	**Error of the mean**
χ_1	RNA-TB	0.3425	2.84×10^{-4}
	RNA-DFT	0.2569	3.50×10^{-4}
	SO3LR	0.3665	1.48×10^{-4}
	MACE-POLAR	0.4723	2.40×10^{-4}
χ_2	RNA-TB	0.4189	3.49×10^{-4}
	RNA-DFT	0.4623	1.07×10^{-4}
	SO3LR	0.6743	1.15×10^{-4}
	MACE-POLAR	0.4185	1.90×10^{-4}
δ_1	RNA-TB	0.3399	4.48×10^{-4}
	RNA-DFT	0.1924	2.64×10^{-4}
	SO3LR	0.2865	2.83×10^{-4}
	MACE-POLAR	0.2650	3.79×10^{-4}
δ_2	RNA-TB	0.3618	3.39×10^{-4}
	RNA-DFT	0.3185	1.72×10^{-4}
	SO3LR	0.5013	2.68×10^{-4}
	MACE-POLAR	0.6654	1.90×10^{-5}
γ	RNA-TB	0.5476	2.93×10^{-4}
	RNA-DFT	0.2976	2.43×10^{-4}
	SO3LR	0.7988	2.04×10^{-4}
	MACE-POLAR	0.3513	3.66×10^{-4}
ε	RNA-TB	0.4179	1.74×10^{-4}
	RNA-DFT	0.2348	2.90×10^{-4}
	SO3LR	0.2498	1.93×10^{-4}
	MACE-POLAR	0.2794	2.39×10^{-4}
ζ	RNA-TB	0.7654	1.00×10^{-3}
	RNA-DFT	0.6971	4.18×10^{-4}
	SO3LR	0.7922	7.20×10^{-5}
	MACE-POLAR	0.8238	9.40×10^{-5}
β	RNA-TB	0.5070	2.14×10^{-4}
	RNA-DFT	0.6836	3.12×10^{-4}
	SO3LR	0.8285	2.73×10^{-4}
	MACE-POLAR	0.8846	1.32×10^{-4}

References

[1] Hayatshahi, H. S., Henriksen, N. M. and Cheatham III, T. E., *Consensus conformations of dinucleoside monophosphates described with well-converged molecular dynamics simulations*, J. Chem. Theory Comput. 14, 3, 1456–1470 (2018).

[2] Richardson, J. S., Schneider, B., Murray, L. W., Kapral, G. J., Immormino, R. M., Headd, J. J., Richardson, D. C., Ham, D., Hershkovits, E., Williams, L. D., et al., *RNA backbone: consensus all-angle conformers and modular string nomenclature (an RNA Ontology Consortium contribution)*, RNA 14, 3, 465–481 (2008).
